# Treatment Needs and Rates of Mental Health Comorbidity in Adolescent Patients With ARFID

**DOI:** 10.3389/fpsyt.2021.680298

**Published:** 2021-07-16

**Authors:** Mark L. Norris, Nicole Obeid, Alexandre Santos, Darcie D. Valois, Leanna Isserlin, Stephen Feder, Wendy Spettigue

**Affiliations:** ^1^Children's Hospital of Eastern Ontario Research Institute, Ottawa, ON, Canada; ^2^Division of Adolescent Medicine, Department of Pediatrics, Children's Hospital of Eastern Ontario, University of Ottawa, Ottawa, ON, Canada; ^3^Department of Psychiatry, Children's Hospital of Eastern Ontario, University of Ottawa, Ottawa, ON, Canada

**Keywords:** avoidant restrictive food intake disorder, service organization, pilot study, retrospective cohort study, multidisciplinary, treatment, comorbidity

## Abstract

The purpose of this paper is to provide a descriptive overview of a single-center ARFID-specific pilot clinic that sought to better understand the specific needs of patients with ARFID including rates of comorbidities, and to gain insight into treatment requirements. A retrospective cohort study was completed on patients meeting criteria for ARFID admitted to a specialized pilot clinic within a tertiary care hospital. Over an 18 month period, a total of 26 patients were assessed and had follow-up data for a 12 month period. Patients presented with heterogeneous manifestations of ARFID and high rates of comorbid mood and anxiety disorders were noted. Treatment plans were tailored to meet individual needs at assessment and over the treatment period. A multidisciplinary approach was most often administered, including a combination of individual therapy, family therapy, medical monitoring, and prescribed medications. Only 30% of patients were treated exclusively by therapists on the eating disorder team. The experiences gained from this pilot study highlight the need for specialized resources for assessment and treatment of patients with ARFID, the importance of a multidisciplinary approach to treatment, and the necessity of utilization of ARFID-specific measures for program evaluation purposes.

## Introduction

Avoidant restrictive food intake disorder (ARFID) is a feeding or eating disturbance introduced in the *Diagnostic and Statistical Manual of Mental Disorders* Fifth *Edition* [DSM-5; (1)] and described in the section entitled “Feeding and Eating Disorders.” Since its introduction, researchers have sought to better understand and describe ARFID [e.g., (2–4)]. Although the incidence of ARFID in community-based populations of children and adolescents has not been well-established, a large Canadian surveillance study reported an overall incidence of 2 per 100,000 children and adolescents aged 5–18 years old (5). Many early studies have reported on patients drawn exclusively from eating disorder (ED) programs and have attempted to understand ARFID in the context of other EDs, including anorexia nervosa [AN; (6–9)]. Emerging research has suggested that the ARFID diagnosis encapsulates a heterogeneous group of patients with varying presentations that may be distinct in etiology (10, 11). This includes a proportion of patients with ARFID who present with histories of lengthy pre-existing feeding disturbances (3). Studies to date support three primary driver of food avoidance which are provided as examples in the DSM-5 (low appetite/ limited interest, sensory-based food avoidance, and fears associated with eating) as well as various presentations of mixed symptoms (12). Compared to patients with other ED diagnoses, multiple studies have found that patients with ARFID are younger than those with AN, more likely to be male, and have higher rates of anxiety (6–8).

Prior to the introduction of ARFID in the DSM-5, program evaluation data demonstrated the existence of a small cohort of older children and adolescents with restrictive feeding behaviors who lacked body image concerns but exhibited significant restricted intake, medical compromise, and comorbid mental health conditions (7). Given the lack of body image concerns and absence of a diagnosis of an ED such as AN or bulimia nervosa (BN), these patients historically received treatment outside of the ED program from a myriad of healthcare providers within the hospital and greater community, including the consultation-liaison psychiatry team and the gastroenterology team, amongst others. As education and research surrounding ARFID as a new disorder in the DSM-5 increased, so too did the number of requests for formal ED assessments for such patients. This resulted in a host of operational questions for our ED program as well as the hospital, including those relating to the ability and suitability of patients with ARFID to be managed by our ED program, mechanism of triage, assessment tools, program evaluation metrics, and treatment provision. With funded resources for patients with AN and BN already limited within our region, creative discussion among healthcare providers has been required to best determine how patients meeting criteria for ARFID can be optimally and efficiently managed.

To better understand the specific needs, rates and extent of comorbidity, and to gain insight into treatment requirements of patients with ARFID, a dedicated clinic for older children and adolescents meeting criteria for ARFID was piloted at our hospital. This paper provides a descriptive overview of this experience and includes both challenges and knowledge gained throughout the study's timeframe.

## Materials and Methods

For the past 20 years, our pediatric ED team has triaged, assessed, and treated patients with severe EDs as evidenced by medical instability, growth compromise, suicidal ideation in the context of an active ED, or substantial ED symptom impact. Our hospital-based multidisciplinary team of ED healthcare professionals provide treatment in outpatient, inpatient, and day hospital settings. Our team members consist of social workers, psychologists and psychiatrists, who provide a combination of FBT, and individual or group CBT, DBT, and motivational therapy; the psychiatrists also prescribe psychotropic medication as required.

Dietary and nutritional treatment is provided by registered dietitians, while adolescent health physicians and a nurse practitioner manage medical care and provide general mental health (MH) support to patients and families. However, patients with chronic feeding disorders have historically been managed by a variety of other hospital-based specialists from varying disciplines (i.e., occupational therapists, behavioral psychologists, pediatricians, etc.), or in the case of infants and children <6 years of age, a dedicated interdisciplinary team of feeding specialists consisting primarily of occupational therapists, psychologists, and a registered dietitian (13). At present, there is no dedicated resource within our region for older children and adolescents with complex feeding presentations that are not linked to underlying body image concerns.

After careful discussion with key stakeholders from various departments within a tertiary care children's hospital in Ontario, Canada (namely from Pediatrics and Mental Health) and with the understanding that resources required to staff and support a “new” multi-disciplinary clinic were generally unavailable, a pilot clinic was nonetheless initiated. The half-day, bi-weekly ARFID clinic was piloted for ~18 months (December 2014 to June 2016) at the hospital, where a senior adolescent medicine physician (MLN) with substantial experience in the assessment and care of patients with mental health concerns, EDs and complex feeding presentations presided over all clinic operations.

Referrals for this pilot program were triaged by the ED team; triage acceptance criteria were set as patients age 10 and older with concerns primarily relating to medical stability [e.g., bradycardia with heart rate <60 beats per minute, postural hypotension, temperature <35.5 degrees oral, or weight <80% estimated treatment goal weight (14) or growth compromise/threat (deceleration or arrest of growth velocity)] secondary to a feeding disturbance or an ED that was not obviously attributable to body image concerns or fear of weight gain.

For patients meeting acceptance criteria into the pilot clinic, all primary assessments were completed by the clinic's overseeing physician. At assessment, a thorough developmental, feeding, nutritional, and psychosocial history was completed using individual and family interviews. Psychological measures using ED-specific questionnaires were utilized in the early months of the clinic but given their limitations and strain on limited psychometric resources, this practice stopped after ~4–6 months. Psychological measures were administered thereafter when either concerns regarding the presence of possible AN were raised during or after initial assessment, in cases where a comorbid MH condition (or MH distress) was present and felt to be interfering with treatment, or if greater clarity regarding the presence of anxiety or mood disorders was required. For the purposes of this study, psychosocial impairment was defined as impairment resulting in distress in psychosocial function as relayed by either patient or caregiver over the course of assessment or during treatment. It should be noted that ARFID-specific measures or inventories were not available during the timeframe that the pilot clinic operated. Laboratory and other diagnostic testing protocols were conducted at assessment on an individualized basis. Treatment was provided to patients on an individual basis and based upon feeding disturbance as well as comorbid MH presentation and needs. The primary physician liaised with other treatment providers to arrange and provide treatment specific to individuals' needs (e.g., members of the ED team as well as those from other disciplines in the hospital). Follow-up care and case coordination was provided by the primary physician over the course of the pilot timeframe.

Data pertaining to patients' assessments and treatment trajectories for the duration of this pilot were gathered via a retrospective chart review by members of the research team. In order to provide a narrative of treatments offered and received, we included only patients with data from both assessment and 6/12 months post-assessment. Data were collected and analyzed using SPSS 24.0. Descriptive and frequency analyses were performed where appropriate. A relatively small sample size precluded any comparison analyses between groups. This study received ethical approval from the hospital's Research Ethics Board.

## Results

Over 18 months, a total of 26 new patients were assessed that had 1-year follow-up data available. Thirty one percent (*n* = 8) presented with a chief complaint of chronic low appetite, or limited interest in feeding, 23% (*n* = 6) presented with an acute history of restriction precipitated by a traumatic feeding or stressful life event (i.e., fears related to an aversive experience), 8% (*n* = 2) presented with a feeding disturbance attributed primarily to longstanding sensory-based food avoidance (i.e., very severe selective eating and/or food neophobia), and 38% (*n* = 10) presented with mixed presentations of at least two of the previously described presentations (four cases each of aversive experience/ sensory and aversive experience/ low appetite, limited interest and two cases of sensory/low appetite, limited interest).

Demographic information as well as illness and treatment specific indicators are provided in Table 1. The overwhelming majority of patients (92%, *n* = 24) were low weight at time of initial assessment. Thirty one percent of patients (*n* = 8) and/or caregivers identified sensory issues related to feeding as a barrier to normalized eating at first assessment (i.e., severe picky eating or food neophobia). Self-reported length of illness was substantially shorter in those with histories of acute restriction secondary to an aversive experience as compared to other presentations. Mental health comorbidity was high in all groups (Table 2). Anxiety was most commonly noted across the entire cohort (*n* = 19, 73.1%) and although not the case for youth with low appetite/limited interest, more than 50% of patients in each of the remaining and mixed groups had a concurrent anxiety disorder. Just over one third (*n* = 9, 34.6%) of the sample were diagnosed with depression with representation across all cohorts. Twenty three percent of assessed patients had a diagnosis of autism or autism-spectrum disorder.

**Table 1:** Demographic and assessment characteristics of pediatric ARFID patients (*n* = 26) admitted to a specialized pilot clinic.

	**Aversive Hx (*****n=*** **6)**		**Sensory (*****n=*** **2)**		**Low appetite, limited interest (*****n=*** **8)**		**ARFID-Mixed (*****n=*** **10)**		**ARFID total (*****n=*** **26)**	
	**Median (SD)**	**Range(min–max)**	**Median (SD)**	**Range(min–max)**	**Median (SD)**	**Range(min–max)**	**Median (SD)**	**Range(min–max)**	**Median (SD)**	**Range (min–max)**
**Assessment**										
Age (years)	15.54 (2.26)	10.76–17.43	12.02 (1.09)	11.77–13.77	15.17 (2.66)	9.49–17.51	13.85 (1.99)	9.82–16.63	13.86 (2.30)	9.49–17.51
BMI (kg/m^2^)	16.03 (2.23)	11.78–18.66	15.82 (1.01)	14.43–16.40	14.86 (2.13)	11.71–17.62	14.48 (2.70)	12.63–20.55	15.07 (2.35)	11.71–20.55
zBMI	−2.06 (1.03)	−3.75–0.64	−0.99 (1.06)	−2.62–0.63	−2.53 (0.97)	−4.05–1.27	−2.57 (1.64)	−3.79–1.11	−2.29 (1.26)	−3.84–1.11
HR (bpm)	73.00 (17.10)	54–112	77.00 (23.76)	73–116	88.00 (25.56)	49–130	81.50 (18.63)	45–111	82.50 (21.29)	45–130
%TGW	79.50 (7.86)	72–97	84.00 (9.54)	83–100	86.00 (5.40)	75–91	86.00 (6.93)	79–100	84.00 (6.46)	74–100
LOI (months)	13.00 (6.28)	4–24	129.00 (31.23)	84–144	60.00 (65.10)	19–182	86.00 (68.68)	3–177	60.00 (62.83)	3–182
**End of treatment**										
Length of treatment (days)	314.0 (190.18)	32–670	693.0 (41.01)	664–722	307.0 (297.62)	40–1,001	425.50 (205.04)	169–804	409.00 (223.64)	132–1001
Age (years)	16.29 (1.95)	12.59–18.30	14.67 (1.52)	13.59–15.74	15.68 (2.24)	12.23–18.04	15.03 (1.77)	11.73–17.10	15.35 (2.00)	11.73–18.30
BMI (kg/m^2^)	19.33 (1.13)	17.12–20.65	16.73 (1.01)	16.01–17.44	16.70 (2.21)	12.49–19.11	19.33 (2.49)	14.28–21.91	17.94 (2.25)	12.49–21.91
zBMI	−0.69 (0.52)	−0.96–0.57	−1.42 (0.90)	−2.06–0.78	−1.40 (1.17)	−4.30–1.03	−0.45 (1.16)	−2.93–1.13	−0.89 (1.07)	−3.61–1.13
%TGW	100.00 (7.39)	81–100	96.00 (5.66)	92–100	97.50 (6.14)	82–100	100.00 (0.00)	100–100	100.00 (4.39)	81–100

*BMI, body mass index; zBMI, standardized body mass index by age and sex; HR, heart rate; %TGW, percent of treatment goal weight; LOI, length of illness prior to hospitalization*.

**Table 2:** Sex, comorbid psychological diagnoses, and treatment objectives for pediatric ARFID patients (*n* = 26) admitted to a specialized pilot clinic.

	**Aversive Hx (*n =* 6)**	**Sensory(*n =* 2)**	**Low appetite, limited interest (*n =* 8)**	**ARFID-Mixed(*n =* 10)**	**ARFID total (*n =* 26)**
	**No. (%)**	**No. (%)**	**No. (%)**	**No. (%)**	**No. (%)**
Female	5 (83.33%)	2 (100%)	5 (62.50%)	4 (40%)	16 (62.50%)
**Comorbidities**					
Mood	3 (50.0%)	1 (50%)	2 (25%)	3 (30%)	9 (34.60%)
Anxiety	6 (100.0%)	2 (100%)	4 (50%)	7 (70%)	19 (73.1%)
Autism	1 (16.67%)	1 (50%)	0 (0%)	4 (40%)	6 (23.1%)
OCD	2 (33.33%)	0 (0%)	0 (0%)	2 (20%)	4 (15.4%)
ADHD	2 (33.33%)	1 (50%)	0 (0%)	3 (30%)	6 (23.1%)
Learning difficulties (has IEP)	1 (16.7%)	0 (0%)	3 (37.50%)	4 (40%)	8 (30.8%)
**Treatment Objectives**					
Weight gain	6 (100%)	2 (100%)	8 (100%)	9 (90%)	25 (96.2%)
Increasing food variety	0 (0%)	2 (100%)	0 (0%)	6 (60%)	8 (30.8%)

*OCD, obsessive compulsive disorder; ADHD, attention deficit hyperactivity disorder; IEP, individualized education program*.

Treatment plans varied according to the chief presenting concerns and the disposition of the patient at time of assessment (i.e., outpatient vs. inpatient setting). Similar to family-based treatment principles for AN, primary treatment goals focused on weight rehabilitation when malnutrition was present, as well as empowering families to take control of their child's nutrition and to increase calories as required. Treatment plans evolved according to identified needs of the patients (see Table 3). Although not formally tracked, the frequency of clinic visits with the treating physician varied in accordance with treatment progress, such as the degree of medical compromise and the progression of weight gain. While half of patients with acute restriction due to an aversive experience required hospitalization due to medical instability or absolute food refusal, only 15% of the remaining patients (including those with mixed presentations) were hospitalized at any point during treatment. Patients requiring admission on account of medical instability exhibited either severe bradycardia, or weights <75% TGW. In all cases the medical instability was felt to be related to malnutrition. Depending on case-specific needs, individual/family therapists were recommended when assistance was required to achieve weight restoration, or if mental health morbidity or oral-motor sensitivities were felt to be impacting feeding-specific goals. Family therapy was most likely to be recommended for those with aversive/acute presentations, whereas varying individual psychological therapies (e.g., cognitive behavioral therapy (CBT) with exposure, behavioral therapies) were utilized in other presentations, such as sensory sensitivity. Only 30% (*n* = 8) of patients received psychological therapy exclusively by ED therapists.

**Table 3:** Treatment modalities used for the management of pediatric ARFID patients (*n* = 26) admitted to a specialized pilot clinic.

	**Aversive Hx (*n =* 6)**	**Sensory(*n =* 2)**	**Low appetite, limited interest (*n =* 8)**	**ARFID-Mixed(*n =* 10)**	**ARFID total (*n =* 26)**
	**No. (%)**	**No. (%)**	**No. (%)**	**No. (%)**	**No. (%)**
**Treatment Modalities Utilized**					
Inpatient admission	3 (50%)	0 (0%)	1 (12.50%)	2 (20%)	6 (23.1%)
Outpatient therapy	6 (100%)	2 (100%)	7 (87.50%)	10 (100%)	25 (96.2%)
**Therapy/Treating Team Members**					
Individual therapy	4 (66.67%)	2 (100%)	2 (25%)	4 (40%)	12 (46.2%)
Family therapy	4 (66.67%)	0 (0%)	1 (12.50%)	3 (30%)	8 (30.8%)
Dietitian	0 (0%)	1 (50%)	3 (37.50%)	7 (70%)	11 (42.3%)
Occupational therapist	0 (0%)	2 (100%)	0 (0%)	1 (10%)	3 (11.5%)
**Medication**					
Atypical antipsychotics	4 (66.7%)	1 (50%)	1 (12.50%)	3 (30%)	9 (34.6%)
Appetite stimulants	3 (50%)	1 (50%)	4 (50%)	3 (30%)	11 (42.3%)
SSRIs	3 (50%)	0 (0%)	2 (25%)	3 (30%)	8 (30.8%)
Benzodiazepines	2 (33.33%)	0 (0%)	0 (0%)	2 (20%)	4 (15.4%)
Dopamine receptor antagonists	2 (33.33%)	0 (0%)	0 (0%)	1 (10%)	3 (11.5%)
SNRIs	1 (16.67%)	1 (50%)	0 (0%)	0 (0%)	2 (7.7%)

*SSRIs, selective serotonin reuptake inhibitors; SNRIs, serotonin-norepinephrine reuptake inhibitors*.

The need for clinical reassessment of treatment goals by the treating physician varied and included such changes as when: a psychological or behavioral therapy was initiated, support by a multi-disciplinary member (i.e., a dietitian) was required, a medication was prescribed, or a change in treatment setting occurred (i.e., patients required admission to hospital or were discharged from hospital; see Table 4). Regarding rates of weight restoration, just over half (54%, *n* = 14) of patients were weight restored by 6 months post-assessment. Of note, the median percentage of treatment goal weight (TGW) attained by the time of discharge from treatment ranged between 96 and 100% across all cohorts (Table 1). Those with mixed presentations were proportionately more likely to still be engaged with clinical services at 1 year after initial assessment.

**Table 4:** Treatment trajectories of pediatric ARFID patients (*n* = 26) admitted to a specialized pilot clinic.

	**Aversive hx (*n =* 6)**	**Sensory(*n =* 2)**	**Low appetite, limited interest (*n =* 8)**	**ARFID-Mixed(*n =* 10)**	**ARFID total (*n =* 26)**
	**No. (%)**	**No. (%)**	**No. (%)**	**No. (%)**	**No. (%)**
**Treatment Reassessment Required**	4 (66.67%)	1 (50%)	4 (50%)	5 (50%)	14 (53.8%)
Time to reassessment (days)^a^	17.00 (42.19)	104.00 (n/a)	377.00 (181.07)	32.00 (107.13)	64.50 (150.77)
**Weight Restoration**	5 (83.33%)	1 (50%)	3 (37.50%)	8 (80%)	18 (69.2%)
Restored at 6 months	3 (50%)	1 (50%)	2 (25%)	7 (70%)	16 (61.5%)
Restored at 12 months	5 (83.33%)	1 (50%)	3 (37.50%)	8 (80%)	17 (65.4%)
Time to weight restoration (days)^a^^,^ ^b^	138.00 (114.26)	104.00 (n/a)	193.00 (145.66)	111.00 (130.56)	153.50 (125.97)

a *Where applicable, values given are expressed as median (SD);*

b *Time to weight restoration was calculated based on patients who achieved weight restoration*.

## Discussion

The objective of this study was to gather knowledge regarding clinical and demographic characteristics, treatment needs and rates of comorbidity through a single-center pilot initiative of a clinic for youth with ARFID. Our experience highlighted several key findings that have helped our hospital better understand how resources can be aligned to best meet the needs of such patients. First, despite the very stringent inclusion criteria set for this initiative, 26 patients with substantial medical and MH comorbidity were included, suggesting a strong need for a clinic or service within our hospital that serves older children and adolescents with complex feeding presentations.

Although our inclusion criteria for acceptance into the pilot clinic were purposefully narrow, our justification was that the high medical needs of patients assessed would almost certainly have resulted in assessment by at least one hospital-based physician or service during the timeframe that the pilot ran. Regarding inclusion criteria, this experience highlighted an important lesson learned relating to our omission of markers of impairment on our triage form. By only accepting referrals with demonstrated evidence of medical instability, we excluded patients that met criteria for ARFID solely on the grounds of psychosocial impairment, even when severe. This has relevance given recent study findings that suggest that the patients with ARFID who are included based primarily on the criterion of psychosocial impairment are often normal weight (15). Although one of our purposes in establishing this pilot clinic was to better understand the scope and complexity of issues that patients with ARFID might present with, our decision to limit inclusion criteria introduced bias into our results. This would be an important consideration for future program and resource planning. Further research is required to clarify how the degree of psychosocial impairment affects illness presentation as well as response to treatment in each of the types of presentations of ARFID.

The patients in this pilot endeavor represented each of the proposed “motivations” or reasons for feeding disturbances outlined in the DSM-5. In keeping with results from other published studies, a high proportion of the sample exhibited more than one driver for feeding impairment (10). Patients with aversive presentations presented with abbreviated course of illnesses and were all diagnosed with anxiety. Higher rates of anxiety in this specific presentation as compared to other ARFID presentations have been noted previously (16). In this sample, patients with low appetite represented the highest proportion of single-symptom presentations. Although other researchers have demonstrated varying proportions of each cohort in clinical samples, further research is required to better understand the prevalence of ARFID presentations in community-based samples (17). Those presenting with primary feeding challenges related to the sensory domain (i.e., limited-variety type) were fewer in number as compared to those with other recognized presentations. Our own research has suggested that those with sensory-based food avoidance (i.e., extremely selective eaters) are less likely to present with markers of medical instability, and as such, would have been less likely to meet inclusion criteria for this pilot initiative (11). Although ED-program specific research in this area remains in its infancy, the hypothesis that those with feeding issues related primarily to sensory difficulties have longer illness courses and low rates of medical instability is supported by multiple reports (11, 17).

Conceptually, if food restriction in ARFID results from lack of appetite, difficulties with sensory characteristics of food, and/or fears related to the act of eating (or combinations of these characteristics), assessment tools and treatment plans need to be able to clearly delineate these specific etiologies, help confirm diagnoses, and meet specific patient treatment needs (18). Given current resource constraints as well as our ED program's very specific approach to the assessment and treatment of patients with severe EDs such as AN and BN, it was unrealistic to assume that treatment options available for patients with AN and BN could meet the breadth of needs of patients with ARFID. Our data supported this assumption, with only 30% of patients exclusively receiving therapy by ED team members. Indeed, the issue of heterogeneous treatment requirements for patients with ARFID was regularly noted for those with mixed presentations. Not uncommonly, patients in this cohort required care by multiple team members across different disciplines, highlighting the necessity of a treatment framework that can adapt to meet the varying needs of patients, as opposed to a “one size fits all” approach. Of interest, a recent systematic review of interventions for severe feeding disorders suggested an intensive multidisciplinary team approach and is in keeping with a best-practice model (19). As eating disorder program adapt to try and meet the needs of patients with ARFID, it will be important to understand how the needs of patients can be best met and as well, how delivered treatments impact overall course of illness (20).

In addition to these considerations, another lesson learned from the study relates to the limited capacity of existing programs to accommodate patients with ARFID. Despite strict inclusion criteria, and a limited assessment window, the pilot initiative resulted in 26 assessments, which represented approximately one third of annual assessments typically completed by our ED program prior to 2020. Given that patients with ARFID are increasingly recognized as requiring prolonged treatment courses and exhibiting high rates of comorbid MH conditions, considerations that relate to any existing program's capacity to accept new referrals cannot be understated. As our hospital currently lacks a dedicated feeding team for children over the age of 6 years, the described pilot project helped bring attention to existing treatment gaps (i.e., sensory-specific feeding interventions for older children and adolescents). It also highlighted the finding that a proportion of patients with ARFID benefit from feeding-specific interventions that involve support, education and gradual exposure to foods deemed unsafe or unappealing. Depending on the specific resources available within institutions, this treatment could be provided outside of the ED team (as was typically the case in this initiative) or by ED team members, assuming that additional training and support could be provided.

Regarding the assessment procedure, in keeping with the findings of Cooney et al. (21), our ED program's psychometric measures were not specific for making a diagnosis of ARFID, and given resource constraints, the practice was stopped. Moving forward, should an ARFID clinic with a program of evaluation be re-established, a number of inventories and measures are now available that would help practitioners better track progress of outcome variables specific to ARFID as treatment progresses as well as (22, 23).

In keeping with previous studies, we demonstrated that patients accepted into this pilot study exhibited high rates of mental health comorbidity, the most notable of which was anxiety. This finding has been demonstrated in numerous other studies to date (12, 16) and provides additional support for the utility of treatments that are mindful of the mental health needs of patients and that provide support and psychoeducation to families. As demonstrated in another recently published study of mental health comorbidity in patients in ARFID, our study also suggests that the specific study of rates of mental health morbidity observed across varying ARFID presentations requires further study (16).

As is the case in the treatment of AN, our initial emphasis in underweight patients with ARFID has been to renourish patients to an optimal treatment goal weight (TGW), with focus on improved nutritional intake and weight gain during the primary phase of treatment. A recent case series highlighted the multi-disciplinary team approach involving family therapy and cognitive behavioral therapy for the care of patients with varying ARFID presentations (24).

Patients that required inpatient hospitalization were discharged to outpatient services with continued emphasis on renourishment and treatment of the feeding disturbance. Although an emerging body of literature has suggested that patients with ARFID can be treated using day hospital models, our existing day treatment program (DTP) was designed for treatment of patients with AN and BN (e.g., includes groups focused on body image). We suggest that further research that explores the utility and optimization of DTP content would help programs such as our own to understand whether modifications should be considered to make this treatment suitable for patients with ARFID.

This pilot initiative also provided some insight into the use of pharmacotherapy as an adjunctive treatment for patients with ARIFD. We demonstrated that patients across all subtypes were treated with a myriad of different medications from different classes, many of which targeted the presence of comorbid MH issues. As an example, atypical antipsychotics (such as olanzapine) were offered to two thirds of the patients presenting with aversive presentations. Although not empirically studied herein, this treatment approach targeted anxiety in an attempt to allow the patient greater ability to accept foods deemed triggering or unsafe (e.g., something that they feared they would choke on, or would cause them to vomit) or to decrease the overall intensity of internal distress. To date, the evidence examining olanzapine efficacy in patients with ARFID has been limited to two case series (24, 25). The use of appetite stimulants (such as cyproheptadine) also requires further study in this population, in order to better understand how these agents might serve to promote nutritional intake and to determine to what extent illness presentation predicts response to these medications (26).

Our outcomes at the 6-month mark suggest that similar to those with other EDs, many patients with ARFID require intensive services for prolonged periods. Those with mixed presentations required care by many multidisciplinary team members, including other medical subspecialists. Although our small sample size precludes statistical comparisons, those with mixed presentations had higher rates of weight restoration at 6 months compared to patients with a single primary influence (e.g., selective eating or low appetite) for the feeding disturbance. It is difficult to know what, if any factors influenced this specific finding (i.e., length of illness), although these patients had more diversified treatment teams. As the number of empirically studied treatments for patients with ARFID evolves, it is likely that researchers will gain a better understanding of how treatments should be dosed and delivered in order to optimize outcomes across respective presentations.

As noted throughout the discussion, this pilot study was not without limitations. We embarked on this initiative as a means of better understanding the types and presentations of patients that might present to a dedicated clinic for patients with severe ARFID. By limiting our inclusion criteria and the study timeframe, we introduced referral bias and limited our total sample. Our results are retrospective and given the lack of diagnostic instruments available at the time of the pilot, standardized and validated measures specific to ARFID were not utilized. Strengths of the study included the fact that a single provider with expertise in ARFID and complex feeding disorders triaged, assessed, and coordinated care for all patients. Also, a consistent interview template was used for all assessments. Remarkably, despite a lack of coordinated programming, almost all patients were weight restored by the time treatment concluded. Although not formally evaluated, chart review suggested that treating the subset of patients with sensory-specific feeding issues (i.e., limited-variety type) proved the most challenging given gaps within the hospital for services specific to the needs of this population. In cases where family-based therapy principles were applied to patients with sensory-specific issues, food selectivity often persisted despite success with weight gain. Most typically, these patients presented with long-standing histories of restrictive eating, dating back to infancy. The lack of a validated instrument that assessed severity and tracked progress in this particular area is another limitation of the study.

This pilot project provided additional evidence that there is an unmet need for coordinated services focused on the assessment and treatment of children and adolescents with complex feeding presentations. Although not formally tracked, a number of referrals were denied because they did not meet our strictly defined inclusion criteria. The small clinic ran at capacity throughout its 18-month operational window. Although we have demonstrated the effectiveness of ED team members to provide treatment to at least a proportion of patients (e.g., using family-based therapy and cognitive therapy), additional resources and the input of other multi-disciplinary care providers outside of the ED team were often required.

Moving forward, it will be important for health providers who assess and provide care to patients with ARFID to have an understanding of the various presentations and comorbidities frequently noted in this disorder. As evidence becomes available, treatment pathways and protocols can be developed that draw upon existing expertise (likely within and outside of ED teams) to serve patients with diverse presentations. As results of pilot treatment studies become available it will also be important to understand how patients with varying presentations respond (27). Given the marked prevalence of overlapping symptoms, and the fact that as many as 50% of patients in clinical samples exhibit more than one presentation of ARFID, utilizing assessment tools and targeted treatments that best match individualized patient needs will be key (10). As noted in Sharp and Stubbs' recent commentary (28), tailored feeding interventions need to account for the behavioral, psychological, organic, oral-motor, and dietary concerns that often co-exist in this population. Our experience with this project has resulted in a better understanding of the need for hospitals to provide a range of specialized, multi-disciplinary resources directed at the triage, assessment, and treatment of youth with ARFID who may present in a variety of ways and with complex mental health needs. This experience also helped highlight specific gaps in services within our hospital, which in turn has resulted in a better multi-disciplinary approach to service coordination for patients (aged 10–17 years) with complex feeding presentations. Although there is much to learn, we remain collectively committed to understanding how limited healthcare resources can be best organized and adapted to meet the needs of patients with complex feeding concerns across the pediatric lifespan.

## Data Availability Statement

Requests regarding data availability can be made by contacting the corresponding author.

## Ethics Statement

This retrospective study was reviewed and approved by the Research Ethics Board at the Children's Hospital of Eastern Ontario.

## Author Contributions

MN, NO, and WS: study conception and design. AS, DV, and NO: data collection. MN, NO, AS, and DV: analysis and interpretation of results. MN and AS: draft manuscript preparation. All authors reviewed the results and approved the manuscript.

## Conflict of Interest

The authors declare that the research was conducted in the absence of any commercial or financial relationships that could be construed as a potential conflict of interest.
